# Supplementary material for: COA5 has an essential role in the early stage of mitochondrial complex IV assembly
Source: Life Sci Alliance. 2025 Jan 8;8(3):e202403013. doi: 10.26508/lsa.202403013 (PMC11711468; doi:10.26508/lsa.202403013)

Figure 3A Source Data

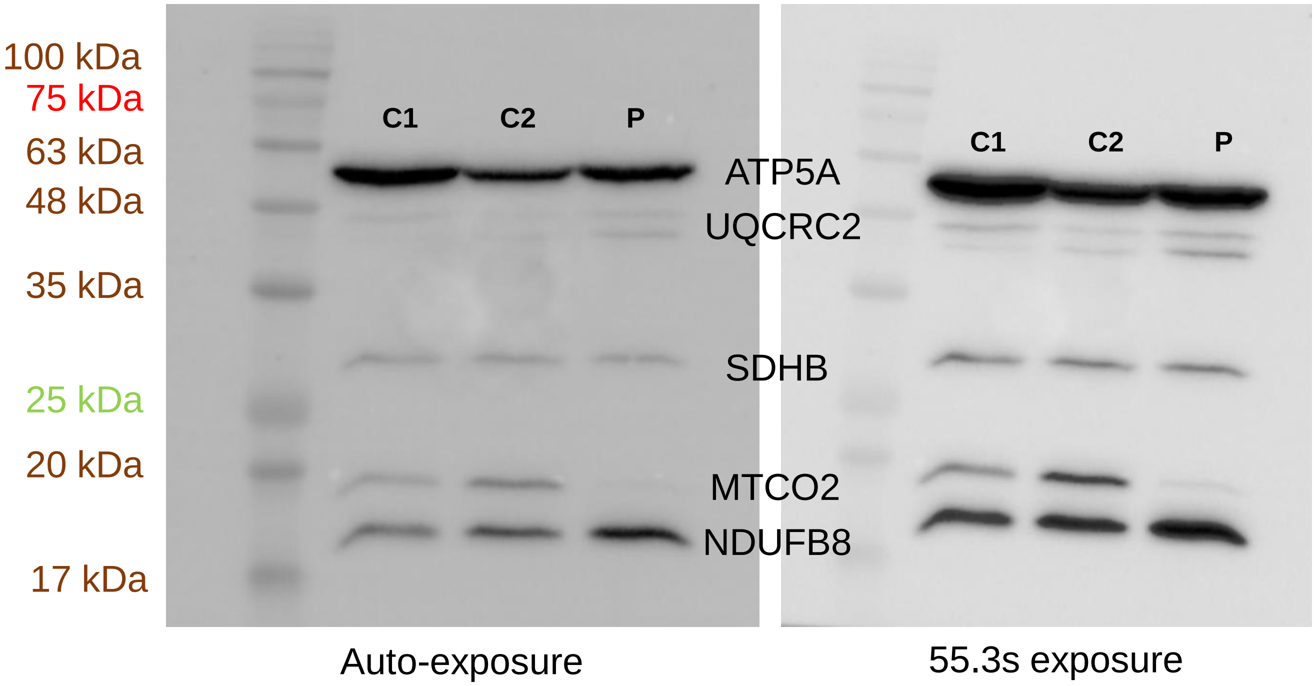

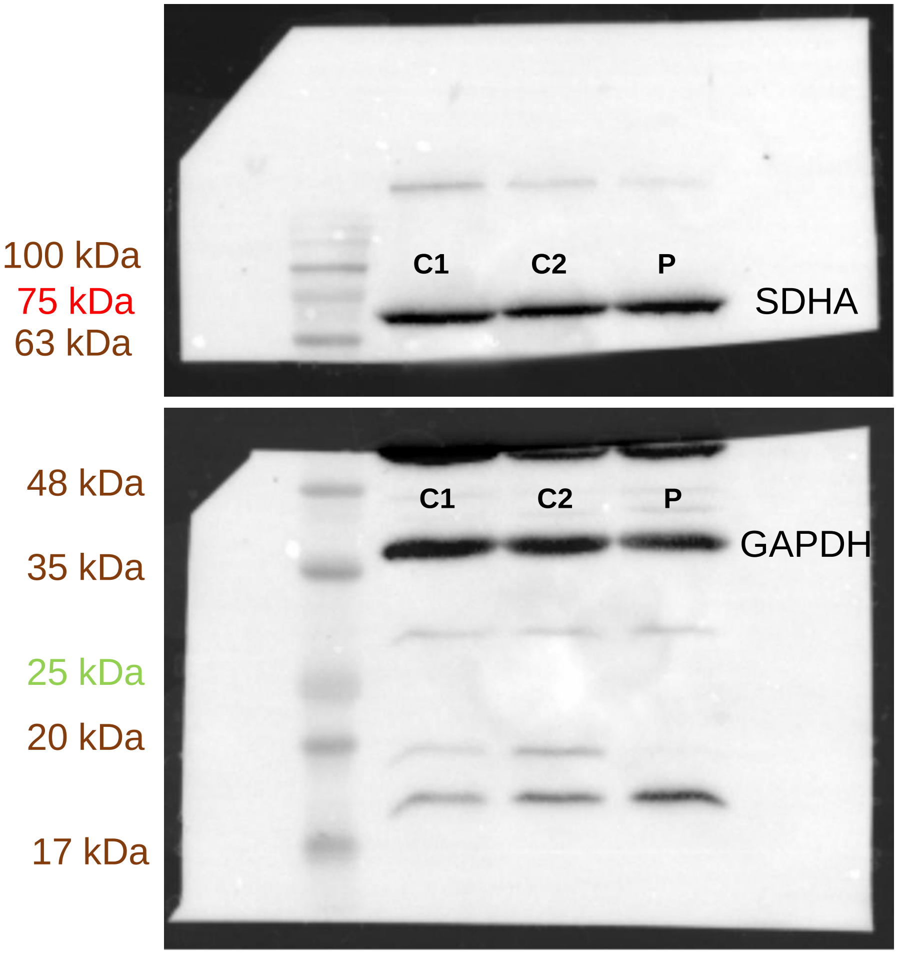

Figure 3B Source Data

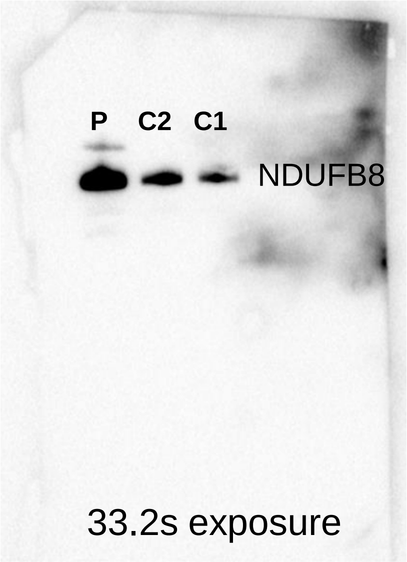

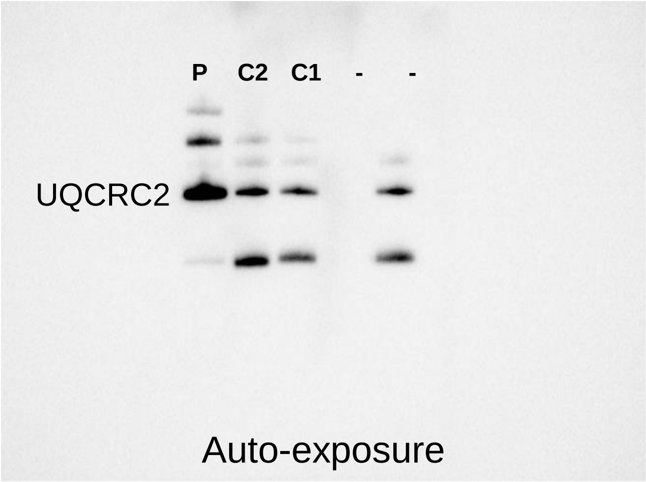

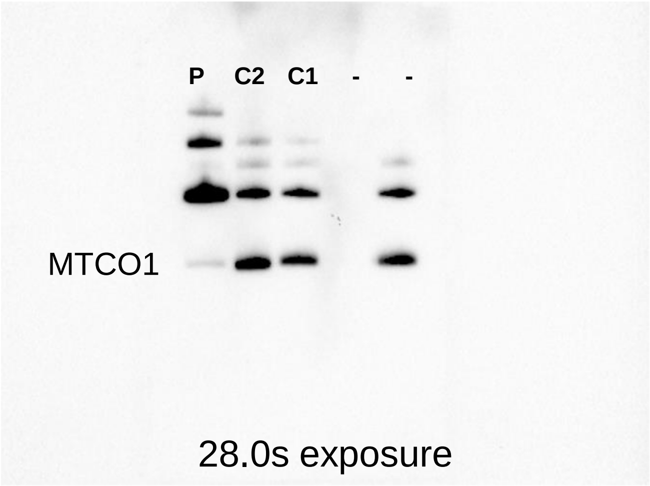

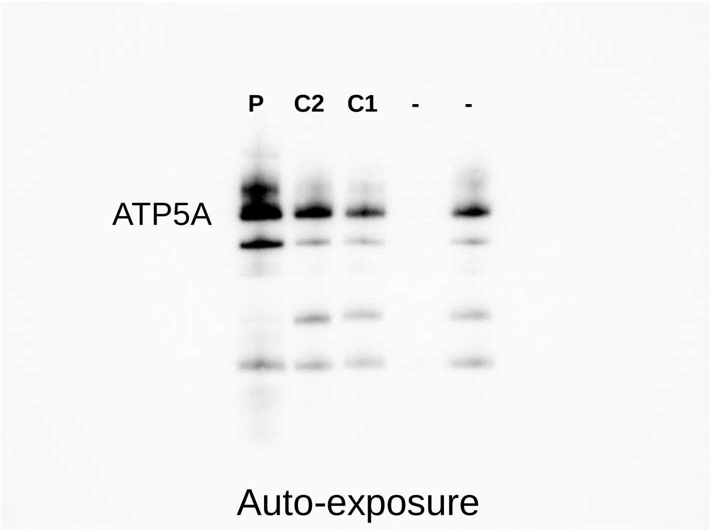

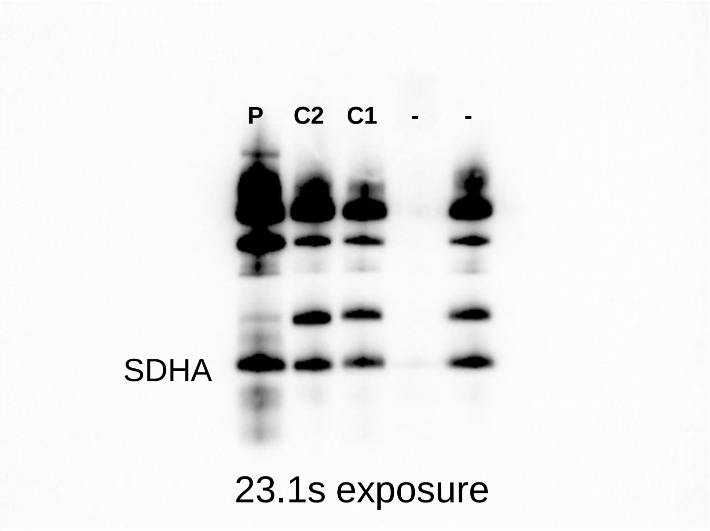

Figure 3C Source Data

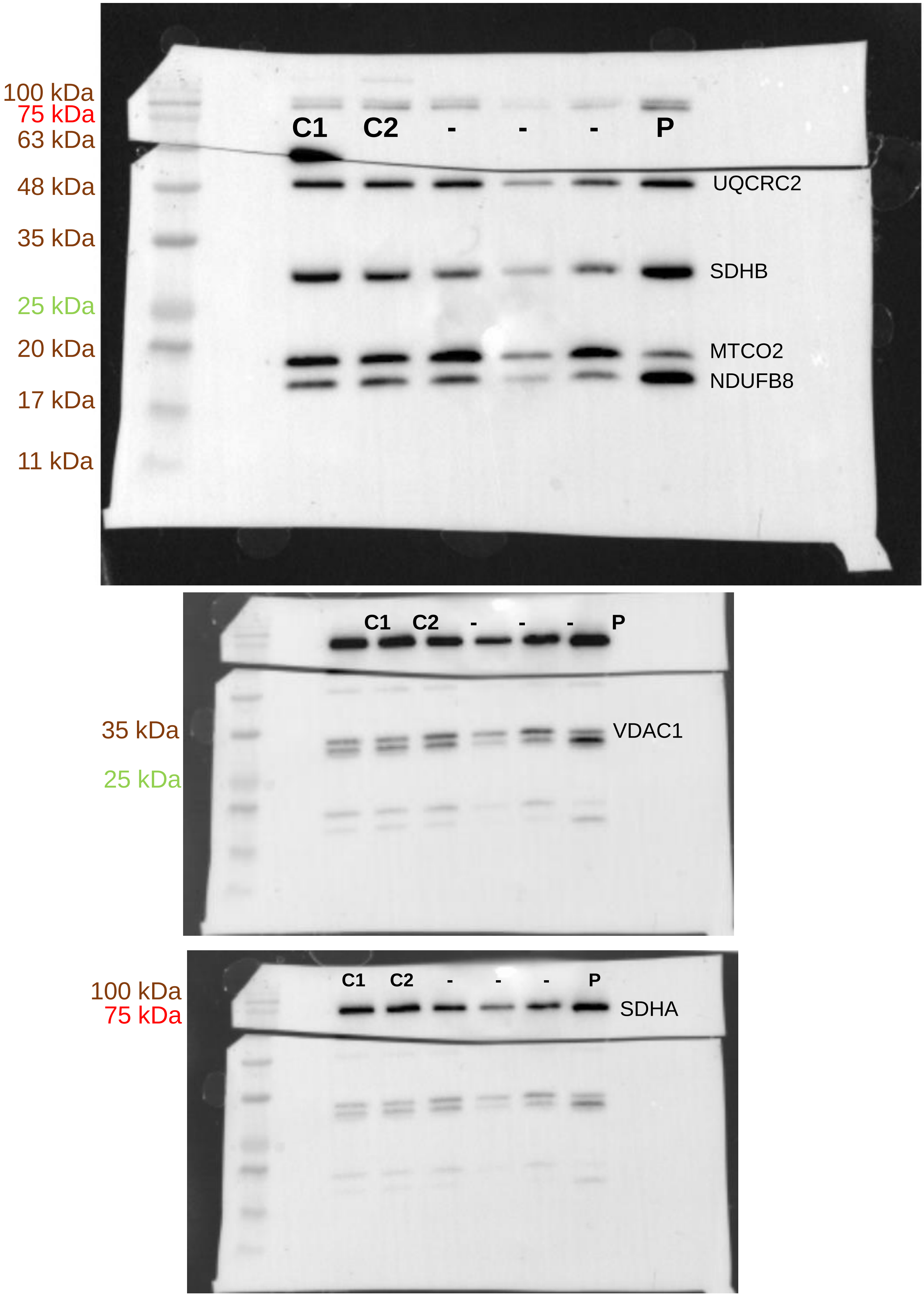

Figure 3D Source Data

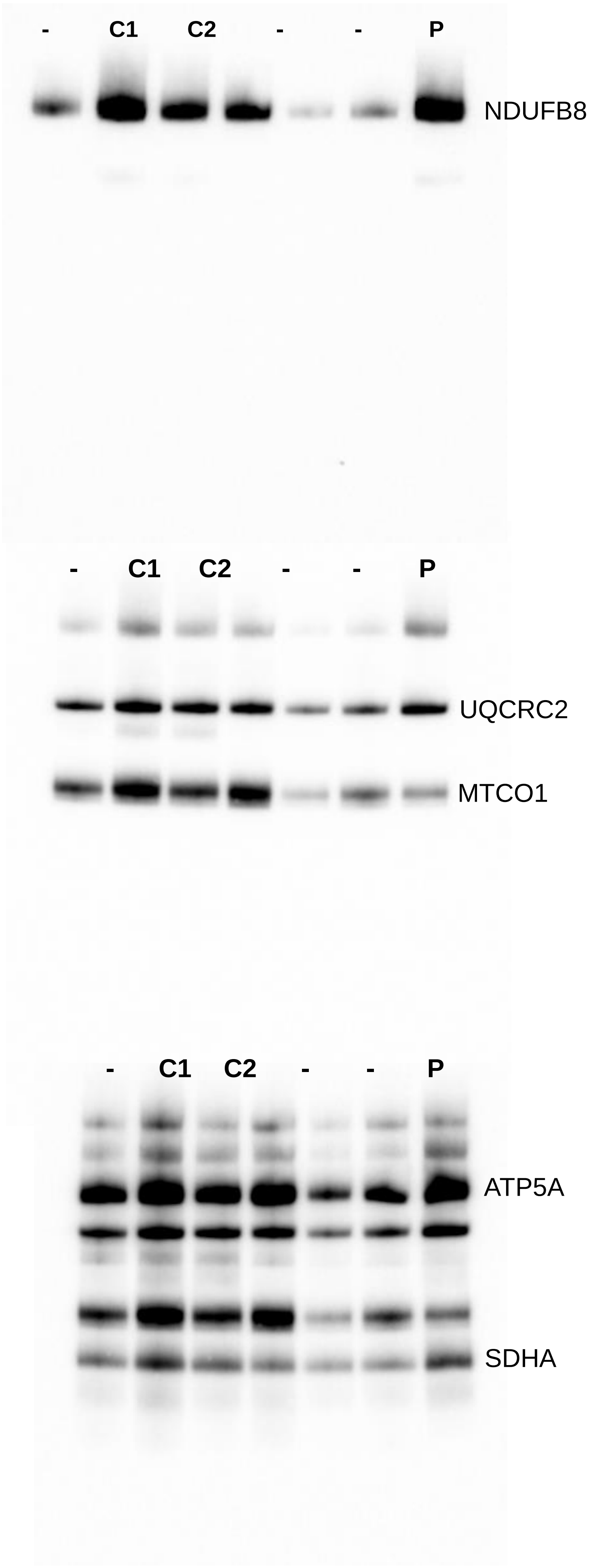

Figure 4A Source Data

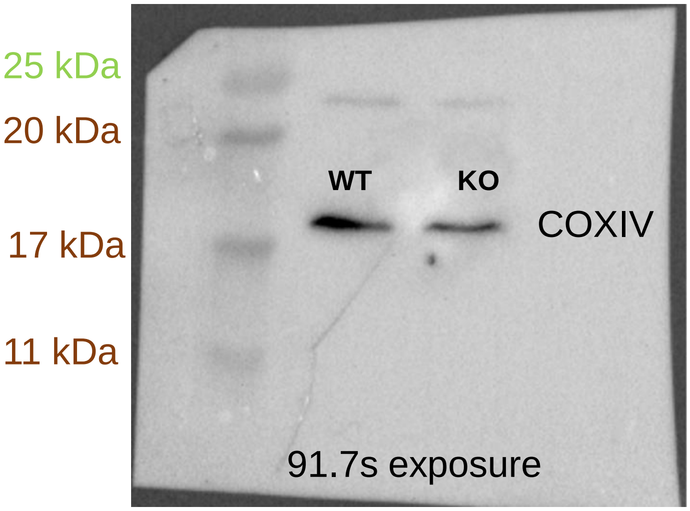

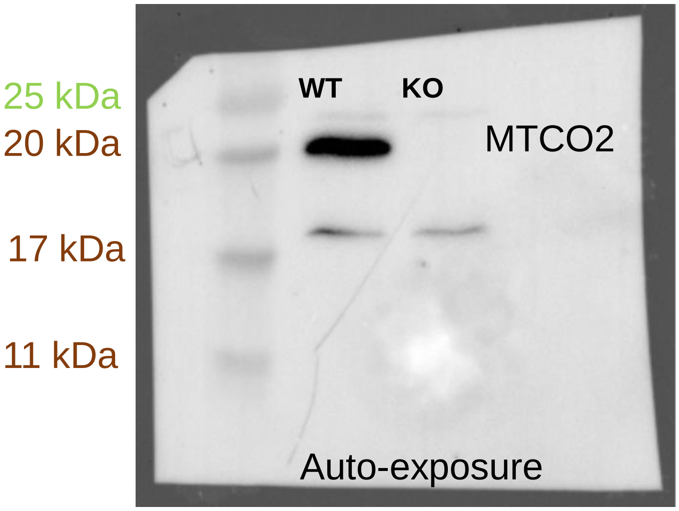

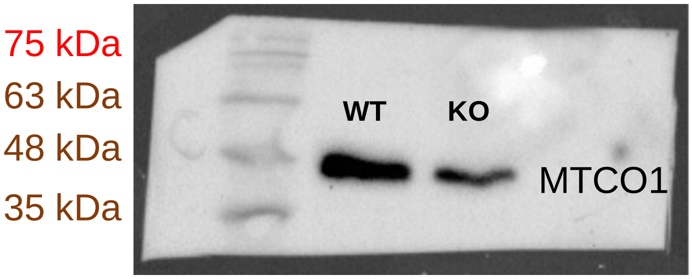

66.5s exposure

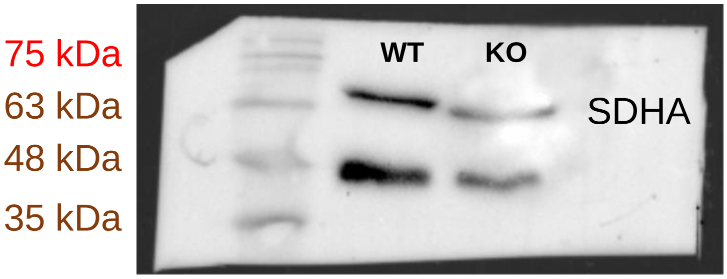

Auto-exposure

Figure 4B Source Data

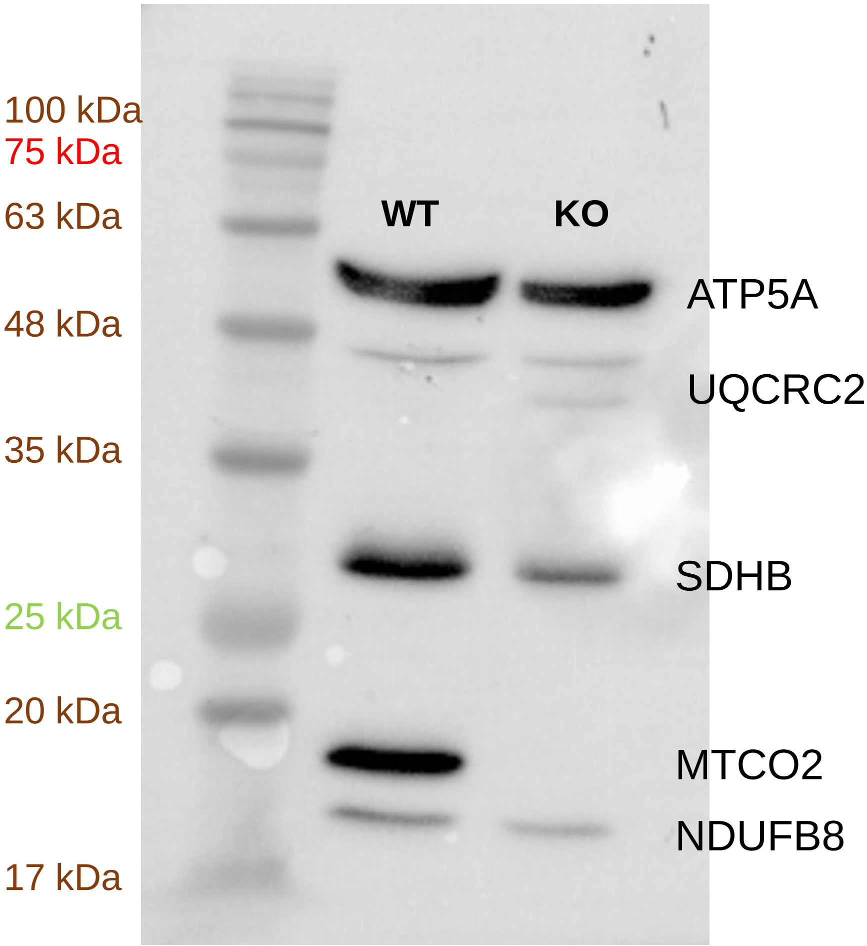

Auto-exposure

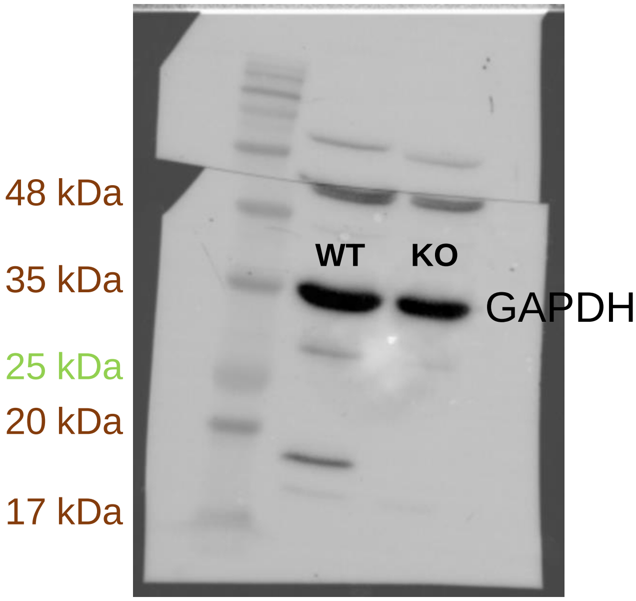

Auto-exposure

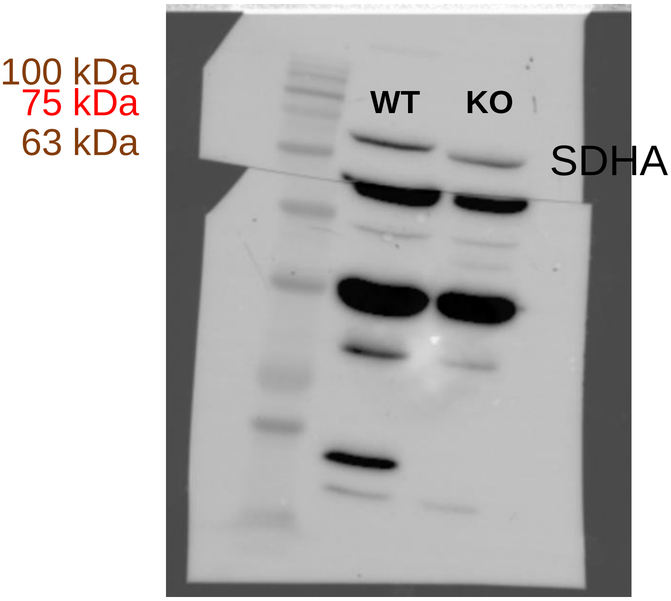

23.0s exposure

Figure 4C Source Data

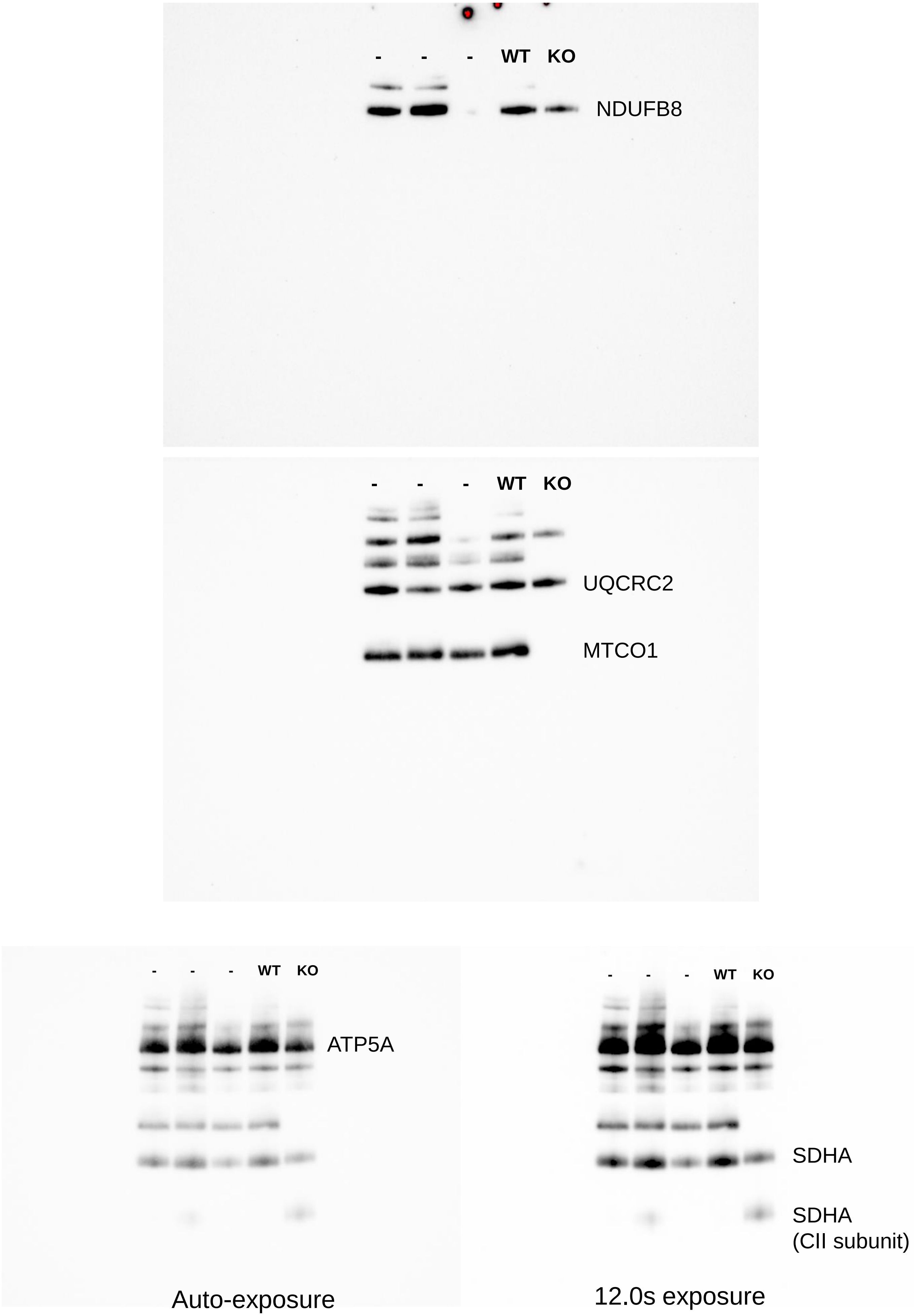

Supplement: Supplementary file 1 [file LSA-2024-03013_SdataF3_F4.pdf]
